# Appreciation

**Published:** 1911-02

**Authors:** 


### Appreciation.

CRYSTAL CITY, TEXAS, January 16, 1911.

*Texas Medical Journal:*

Please place us on your list for the "Red Back," and send us January number. Things are growing dull with us, and we want to be lifted up. The "Red Back" is the only medicine for us.

Yours truly,

DRS. WILLIAMS & MARTIN,

Crystal City, Texas.

---

THORNTON, TEXAS, January 12, 1911.

*My Dear Doctor Daniel:*

Move my subscription up eighteen months, and also send me "The Strange Case of Dr. Bruno." I enjoy the "Red Back" more than any paper I get. It is always fresh; always new. There is only *one* "Daniel."

May your life be spared many years yet, that you may adorn the sanctum of the *best medical journal in the South.*

Sincerely,

W. A. BEDFORD, M. D.

---

Doctor, I congratulate you upon the entertaining manner in which you conduct your most excellent JOURNAL.

With kindest regards,

Very sincerely yours,

J. J. TAYLOR,

Editor *Medical Council,* Philadelphia.

